# Template-Guided Autogenous Tooth Transplantation Using a CAD/CAM Dental Replica in a Complex Anatomical Scenario: A Case Report

**DOI:** 10.3390/dj13070281

**Published:** 2025-06-23

**Authors:** Michael Alfertshofer, Florian Gebhart, Dirk Nolte

**Affiliations:** 1 Department of Oral and Maxillofacial Surgery, Ludwig-Maximilians-University Munich, 80539 Munich, Germany; 2 Private Orthodontic Practice, 80331 Munich, Germany; labor@kfo-gebhart.de; 3 Private Oral and Maxillofacial Surgery Practice, 81377 Munich, Germany

**Keywords:** autogenous tooth transplantation, digital workflow, 3D printing, tooth replica, periodontal ligament, template-guided surgery

## Abstract

**Background:** Autogenous tooth transplantation is a valuable option for dental rehabilitation, particularly in young patients. Template-guided approaches, using 3D-printed replicas of donor teeth, have recently emerged as a method to increase precision and reduce extraoral time—two critical factors in maintaining periodontal ligament (PDL) vitality, which is essential to improve long-term outcomes. **Methods:** This report presents the case of a 12-year-old patient who underwent autotransplantation of tooth 18 to the site of tooth 75, which exhibited ankylosis. Patients exhibiting unfavorable root anatomy and morphology, systemic conditions, or completed root development were not considered for this technique. A patient-specific donor tooth replica was digitally designed and 3D-printed via CAD/CAM manufacturing to preoperatively shape the recipient site. The transplanted tooth 18 was then inserted with an extraoral time of less than one minute and subsequently stabilized using a flexible titanium trauma splint (TTS). **Results:** Longitudinal clinical and radiographic follow-up over 12 months confirmed favorable healing without signs of complications. **Conclusions:** This case illustrates the practical advantages of a fully digital, template-guided workflow in managing anatomically complex cases.

## 1. Introduction

Autogenous tooth transplantation is a biologically favorable treatment for dental rehabilitation, especially in children and adolescents with missing or non-restorable teeth due to trauma, agenesis, or caries. Compared to implants, autotransplanted teeth preserve alveolar bone, maintain proprioceptive function, and, in cases of incomplete root formation, can continue to develop [1,2]. Despite these advantages, conventional autotransplantation techniques face challenges such as prolonged extraoral time and potential PDL trauma during repeated trial placements [3,4]. To overcome these limitations and enhance periodontal ligament cell viability, besides improving the surgical technique, adjunctive methods such as the application of platelet-rich fibrin (PRF), autologous growth factors, and biologically active graft materials have been explored in the literature, aiming to promote early revascularization, reduce inflammation, and support regenerative healing in the transplanted site [5,6,7].

Prolonged extraoral time and PDL trauma increase the risk of complications such as ankylosis or root resorption in the postoperative course. Template-guided tooth transplantation using digitally designed and 3D-printed donor tooth replicas offers a precise, cost-efficient, and minimally invasive alternative [8]. The replicas allow for accurate shaping of the recipient socket before donor tooth extraction and insertion, reducing intraoperative handling and extraoral time. Studies suggest that extraoral times below three minutes significantly improve outcomes [9,10]. The use of computer-aided design (CAD) and additive computer-aided manufacturing (CAM) technologies enables clinicians to virtually simulate the transplantation and assess anatomical limitations preoperatively, thereby enhancing safety and predictability. Moreover, these techniques facilitate interdisciplinary planning by providing visual tools that can be shared between orthodontists, surgeons, and restorative dentists, enabling these stakeholders to align treatment goals, coordinate procedural steps, and support comprehensive, patient-centered planning. This digital approach also allows for simulation-based decision making as well as improved patient education through visual aids. Although formal cost-effectiveness analyses of template-guided tooth autotransplantation are limited, early evidence suggests that template-guided techniques—while requiring additional upfront costs for imaging and 3D printing (e.g., ~USD350 for a printed guide in Taiwan)—this technique may reduce overall treatment cost and burden by shortening surgery times and lowering complication rates. For instance, in one adult cohort, guided transplantation halved the need for post-operative root canal therapy (59% vs. 92%) and achieved extraoral times of less than one minute compared to conventional techniques [11]. Despite these clinical advantages, global adoption remains limited. To date, no large-scale data exist on prevalence or routine use across general practice.

This case report presents a fully digital, template-guided autotransplantation in a 12-year-old patient with complex anatomy and an ankylosed retained deciduous molar. We aim to underscore how a digital workflow can not only improve surgical predictability and outcomes but also support treatment planning in anatomically complex pediatric cases where conventional techniques may be limited. It must be noted that the technique presented herein is best suited for patients with favorable donor tooth morphology, incomplete root formation, and sufficient alveolar bone volume; cases with advanced root development, periapical pathology, or systemic conditions affecting healing should be treated using a different approach.

## 2. Case Presentation

### 2.1. Clinical Examination and Diagnosis

A 12-year-old patient presented for evaluation of a retained tooth 75 in the lower left quadrant. Clinical and radiographic examination revealed ankylosis of the deciduous molar. This was confirmed clinically by the lack of physiological mobility and radiographically by the obliteration of the periodontal space. Due to the patient’s age and ongoing skeletal development, autotransplantation was considered the most suitable option for site preservation and functional rehabilitation. Preoperative CBCT analysis confirmed sufficient height and width of the alveolar ridge, without signs of periapical pathology (e.g., osteolysis) or unfavorable anatomy of the donor and recipient site. Tooth 18 (upper right third molar) was selected as the donor tooth, due to its incomplete root formation and overall favorable morphology for transplantation. Importantly, the donor tooth showed a root development stage of approximately half completion, which is ideal for pulp revascularization and long-term survival. The treatment decision was made collaboratively between the surgical and orthodontic teams to ensure that occlusal and space requirements were met in the long term. Comprehensive patient and parent counseling was conducted, including discussion of risks, benefits, and alternative treatments such as space closure or implants at a later age. Due to the nature of this study as a single routine clinical case conducted within the standards of care without any experimental nature, formal approval by an ethics committee was not required. Informed consent for the procedure and the publication of anonymized clinical data and radiographic images was obtained from the patient’s legal guardians prior to commencing the procedure.

### 2.2. Digital Workflow: Planning and Replica Fabrication

CBCT imaging was performed to capture detailed three-dimensional views of both the donor region and the recipient site. The scan enabled a precise assessment of tooth 18, including root curvature, apical anatomy, and spatial relationships with surrounding structures. Using OnyxCeph^®^ software (Version 3.2.200), the donor tooth was segmented and virtually extracted. This was achieved through semi-automatic thresholding and manual refinement of the segmentation mask to remove adjacent anatomical structures such as the alveolar bone, artifacts, or adjacent teeth. Minor challenges during the segmentation process included the precise isolation of the donor tooth from overlapping anatomical structures. Despite this, the final replica showed excellent clinical fit and was successfully used for guided socket preparation. Parameters such as root length, crown dimensions, and potential anatomical constraints were accounted for during digital modeling. After virtual extraction, the 3D model of the donor tooth was exported as an STL file.

**Figure 1:**
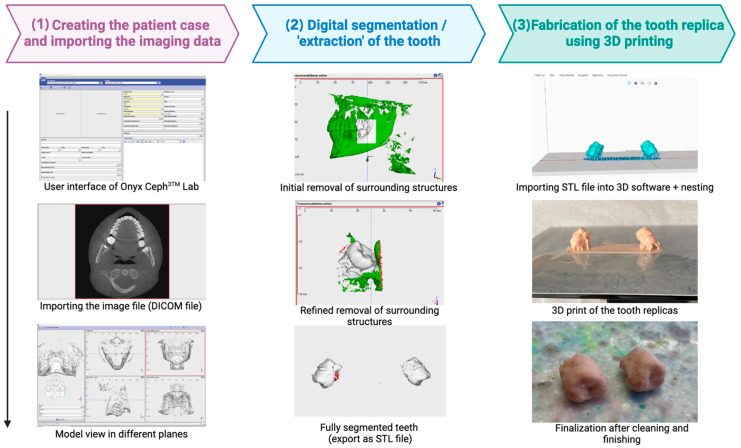
Schematic overview of the digital workflow for the fabrication of tooth replicas in template-guided autogenous tooth transplantation.

The resulting STL file was then sliced using the Anycubic software to optimize its shape for 3D printing. Digital pre-visualization of the replica within the CBCT scan allowed for virtual try-ins and minor adjustments before printing, helping ensure a better fit. To ensure adequate dimensional accuracy, the in-clinic 3D printer was pre-calibrated according to standard manufacturer guidelines. The dental replica was printed using fused deposition modeling (FDM) due to its high precision, cost-effectiveness, and in-clinic availability. After printing, the replica underwent manual post-processing, including support removal and surface smoothing. Special attention was given to refining the apical and cervical areas to ensure dimensional fidelity in the socket preparation phase. Figure 1 illustrates the digital workflow applied in this case, from image acquisition to replica fabrication. This stepwise process provided not only a planning tool but also enhanced communication with the orthodontic team regarding potential adjustments to the eruption and alignment plan. Dimensional accuracy was verified by comparing the printed replica to the original STL file and by evaluating clinical fit during recipient socket preparation. The chosen FDM technology, while accessible and cost-efficient, is subject to known limitations such as potential material shrinkage and reduced surface resolution. These limitations were mitigated by high-resolution slicing (0.1 mm layer height), print orientation optimization, and manual refinement during post-processing. Nevertheless, minor deviations in surface texture and dimensional accuracy might occur and should be considered when translating digital plans into clinical use. Prior to surgical use, the donor tooth replica was chemically disinfected via immersion in a 2.5% glutaraldehyde solution for 20 min in accordance with disinfection protocols for heat-sensitive materials. This method was selected due to its compatibility with FDM-printed models, which cannot tolerate high autoclave temperatures without deformation.

### 2.3. Surgical Workflow

The surgical procedure was carried out under local anesthesia using articaine with epinephrine 1:100,000 to ensure hemostasis. Atraumatic extraction of tooth 75 was performed using a periotome and fine luxators to minimize trauma to the alveolar bone and preserve the buccal and lingual cortical plates, which are critical for transplant stability. Due to the ankylosis, gentle force was required with a dental elevator, and small bone fragments were removed as necessary. The residual socket was thoroughly curetted and irrigated with sterile saline to remove granulation tissue and debris. After extraction, a sterile physiologic saline bath was prepared adjacent to the surgical field as a precautionary measure, although interim donor tooth storage was ultimately not necessary due to the overall quick workflow.

The 3D-printed replica of tooth 18 was then inserted into the recipient site of the previously extracted tooth 75 to guide alveolar socket preparation. Sequential shaping was performed with round burs (2.3 mm to 3.5 mm) and piezoelectric instruments, under copious saline irrigation to prevent overheating and contamination. Socket preparation was carried out to approximately 90% of the estimated root length based on the CBCT planning, deliberately leaving the apical region untouched to form a physiological apical gap and thereby avoid injury to developing structures and promote spontaneous bone healing. Tactile feedback was used to assess proper fit, and the recipient site was adjusted to allow a slight apical gap (1–2 mm), minimizing the risk of pressure-induced root damage.

To ensure soft tissue integration, mucosal flap management was initiated at this stage. A small envelope flap was released without vertical incisions, preserving vascular supply while allowing for tension-free closure over the transplanted root surface.

Once the socket had been fully prepared, attention turned to donor tooth extraction. Tooth 18 was carefully removed using a microsurgical flapless approach and atraumatic luxation with fine periotomes and special extraction forceps. Special emphasis was placed on avoiding any trauma to the root surface or the periodontal ligament during the extraction process. The tooth was inspected visually for integrity and immediately transferred to the prepared socket without any delay. No temporary storage or disinfectant immersion was used, allowing for an extraoral time of less than one minute.

The donor tooth was positioned within the recipient site with slight final adjustments to ensure proper seating. Previously, sharp bone edges of the alveolar socket were smoothed with round burs to prevent mechanical interference with the root surface and thus trauma to the PDL. The dimension of the apical gap was confirmed intraoperatively through a combination of tactile feedback during replica fitting and visual inspection for unobstructed seating of the donor tooth. Occlusion was checked to confirm that the transplanted tooth was in infraocclusion and free of any functional loading. This infraocclusion was intentional to avoid early occlusal loading and is expected to self-correct over time due to continued eruption and skeletal growth. If residual infraocclusion persists at follow-up, orthodontic intervention (e.g., extrusion therapy) will be considered.

A flexible orthodontic splint (e.g., TTS wire) was applied to stabilize the transplant. A 0.4 mm diameter twisted titanium splint was passively bonded to adjacent teeth using light-cure composite resin, allowing for physiological tooth movement and minimizing the risk of ankylosis. TTS was chosen due to its proven clinical stability and ease of passive application, and superior control over physiological mobility in growing patients.

Postoperative care included prophylactic antibiotics (amoxicillin 50 mg/kg/day, divided doses for 5 days) and soft dietary instructions for three weeks. Antibiotic coverage was administered to reduce the risk of postoperative infection and ultimately support early periodontal healing.

Brushing near the transplant site was deferred for one week, and the patient was instructed to avoid any biting or loading on the affected quadrant.

Sutures were not deemed necessary due to tension-free soft tissue adaptation. No signs of hematoma, infection, or wound dehiscence were observed during the early postoperative period.

**Figure 2:**
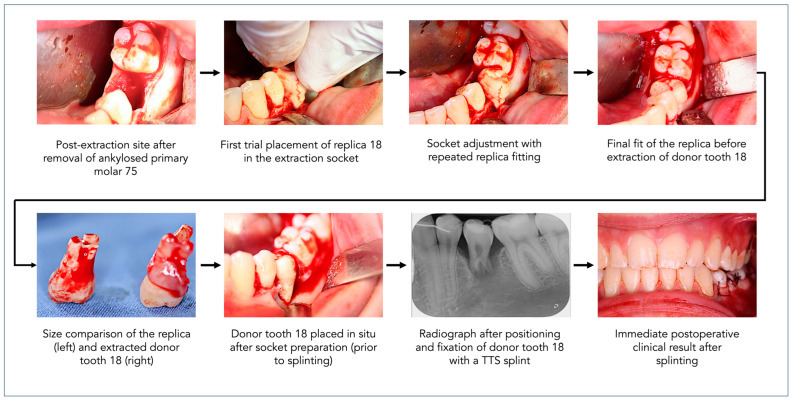
Stepwise surgical workflow illustrating the use of a 3D-printed donor tooth replica to aid in recipient site preparation during autogenous transplantation of tooth 18 to site 75.

Figure 2 summarizes the surgical workflow using the replica for socket preparation.

### 2.4. Follow-Up and Outcomes

The transplant remained stable and exhibited physiological mobility at the 4-week follow-up. Radiographs showed no signs of root resorption or ankylosis. No color changes or other clinical symptoms were reported by the patient. The splint was removed after 3 weeks as planned, and the tooth demonstrated appropriate functional integration without signs of mobility beyond expected physiological parameters. Oral hygiene was consistently maintained, and no plaque accumulation or food impaction was observed at the transplant site. By 6 months, the transplant had successfully integrated, with healthy surrounding bone and periodontal tissues. The patient reported high satisfaction in terms of aesthetics and function. A vitality test was deferred due to the ongoing root formation. The radiographic image at 6 months demonstrated continued apical development, indicating adequate healing and likely pulp revascularization. No periapical radiolucencies or periodontal pockets were noted. Clinically, the gingival margins showed excellent adaptation without signs of inflammation, gingival recession, or scarring. The soft tissues remained stable, and probing depths were within physiological limits.

Longer-term follow-up at 12 months has been scheduled to monitor root maturation and occlusal integration. To limit radiation exposure in pediatric patients, a CBCT scan is planned at that time only if clinical or radiographic findings warrant further investigation. A CBCT scan is planned at that time only if clinical or radiographic findings warrant further investigation. Continued surveillance will focus on apex closure, pulp vitality, and long-term periodontal support. The standard follow-up protocol after tooth autotransplantation in our clinic includes visits at 4 weeks, 6 months, and 12 months postoperatively, followed by visits once a year to monitor root development, periodontal stability, and long-term functional integration. To ensure long-term occlusal stability and compensate for potential post-eruptive wear, a conservative restoration such as a tabletop or partial coverage crown can be considered once root development and periodontal stability are complete.

**Figure 3:**
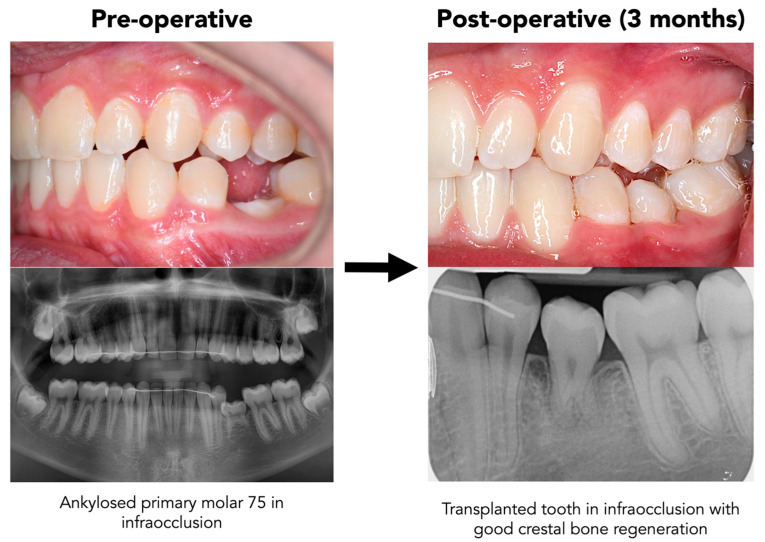
Clinical and radiographic comparison before and after autogenous transplantation of tooth 18 to site 75, demonstrating successful integration and preservation of periodontal structures.

Figure 3 compares the clinical and radiographic situation before and after the transplantation.

## 3. Discussion

Template-guided autotransplantation has gained recognition as a precise and efficient technique. A systematic review by Verweij et al. analyzing 19 studies found survival rates between 95.5% and 100%, and success rates from 80% to 91.1%. These favorable outcomes were attributed to significantly reduced extraoral times, which are essential to maintain PDL vitality [8]. In contrast, a meta-analysis by Almpani et al. on conventional techniques reported a pooled survival rate of 92.2% and a success rate of 85.4%, with higher variability. Failures were largely due to pulp necrosis (34.3%), root resorption (10.4%), and ankylosis (6.2%). Yet, it needs to be noted that although pulp necrosis accounted for 34.3% of reported failures, it does not necessarily constitute clinical failure if managed appropriately with root canal treatment.

Teeth with open apices fared significantly better than fully developed roots due to the former’s potential for revascularization and improved healing in the transplanted site. Moreover, rigid splints were associated with poorer outcomes [1].

The decision to perform root canal treatment on autotransplanted teeth is highly dependent on the respective root development and clinical follow-up findings. Immature donor teeth with incomplete root formation—as presented herein—generally show a great propensity for spontaneous pulp revascularization, provided that trauma to the PDL is minimized and extraoral time is brief. In such cases, prophylactic endodontic treatment is not routinely indicated. Conversely, teeth with fully developed roots have a significantly lower chance of revascularization and are often treated electively with root canal treatment within 2–4 weeks postoperatively to prevent pulp necrosis and subsequent complications, including root resorption. Clinical indicators such as persistent pain, swelling, or periapical radiolucencies during follow-up would also necessitate endodontic intervention. However, unnecessary or premature RCT in immature transplants may impair apical development and long-term survival [12,13]. Therefore, a tailored, case-by-case approach is essential, guided by radiological tooth development and longitudinal clinical assessment.

Short extraoral time was a consistent predictor of success. Andreasen et al. found that PDL healing remains possible if extraoral time is kept below 18 min [14,15]. Shahbazian et al. (2013) [16] compared conventional and template-guided techniques and reported extraoral times under one minute with the template-guided autotransplantation, compared to 3–10 min with traditional methods. Repositioning attempts were also reduced (0–3 vs. 4–7), and overall surgery time was significantly shortened, with 30–45 min vs. 40–90 min [17]. The current case corroborates these findings, with rapid transplant placement and no signs of PDL trauma or resorption at follow-up. Moreover, the digital workflow allowed accurate prediction of spatial constraints, helping avoid root collisions with cortical bone and optimizing flap design. As outlined above, the authors chose the template-guided autotransplantation due to its evident clinical advantages. In similar clinical situations, treatment alternatives include conventional freehand autotransplantation, orthodontic space closure, maintenance of the ankylosed tooth until skeletal maturity, or delayed implant placement. However, each technique shows inherent limitations that make it unsuitable for the present case. Freehand techniques are associated with longer extraoral times, with a greater extent of PDL trauma, and therefore less predictable outcomes, while space closure or prolonged retention of an ankylosed tooth may compromise alveolar bone development and ultimately occlusion. Implants are contraindicated at this age due to ongoing skeletal growth. Our aim was to preserve the alveolar ridge, maintain proprioceptive function, and enable functional and esthetic rehabilitation with minimal biological trauma—advantages that are specifically supported by the template-guided digital workflow [17,18]. In addition to the refined surgical technique (e.g., via 3D-printed replicas and surgical templates), several adjunctive strategies have been proposed to enhance PDL preservation and improve healing outcomes. For example, the use of PRF has demonstrated promising results in promoting early vascularization and reducing postoperative inflammation [19,20]. Emerging approaches also explore the role of bioactive scaffolds and coatings in enhancing the viability of PDL cells [21]. Future protocols may benefit from a multimodal approach combining surgical precision with biologically supportive measures to further reduce complications such as root resorption or ankylosis.

This case is notable for two specific reasons. First, the recipient site presented a unique anatomical challenge due to the ankylosed retained primary molar and partial infraocclusion, requiring customized planning and careful socket preparation. The successful resolution of this scenario highlights the value of template-guided approaches in overcoming complex pediatric anatomical conditions.

Second, the use of a low-cost, chairside FDM-printed replica demonstrates that advanced digital workflows can be implemented in resource-conscious clinical settings. Open-source software and centralized planning services could further reduce local infrastructure demands. While SLA or PolyJet printers have been reported to provide higher accuracy [22,23], this case illustrates that FDM technology—when combined with proper planning and digital designing—can enable safe and efficient transplantation. This opens the door for broader clinical adoption, especially in smaller practices or hospitals.

Template-guided autotransplantation holds considerable potential not only as a clinical innovation but also as an educational tool, offering less experienced surgeons a structured, reproducible approach that enhances technical precision. By reducing intraoperative variability, the technique facilitates safer learning curves and could help standardize training. Even for seasoned clinicians, the workflow may increase efficiency and consistency in anatomically complex or time-sensitive cases such as the one presented herein.

Despite its promise, the current body of evidence is still evolving. Most available data stem from case reports and small series, highlighting the need for larger, well-designed randomized controlled trials with extended follow-up to fully establish the clinical and educational value of this technique. A potential limitation hindering the wide adoption in clinical practice lies in the equipment-intensive nature of the procedure as well as the lack of standardized protocols for digital tooth replica generation, such as segmentation parameters, printer calibration, and material selection. As digital workflows become more widely adopted, the absence of validated, reproducible guidelines may lead to inconsistent clinical outcomes across centers. Future studies should aim to establish consensus-driven protocols that ensure cross-platform compatibility, optimize dimensional accuracy, and define quality control benchmarks. Emerging technologies such as intraoperative navigation—while not yet standard in autotransplantation—could further refine surgical precision and reduce operator dependency, opening new horizons for template-guided autotransplantation in both clinical and educational settings.

## 4. Conclusions

Template-guided autogenous tooth transplantation represents a significant evolution in clinical technique, improving accuracy, reducing extraoral time, and enhancing outcomes. The use of 3D-printed replicas allows for precise socket shaping and minimized PDL trauma. This case further supports the integration of digital tools across the diagnostic, planning, and surgical phases of autotransplantation, especially in complex pediatric cases. Future advancements may include AI-assisted planning, improved biomaterials for replicas, and dynamic navigation technologies for intraoperative guidance. Large-scale clinical studies are needed to assess long-term outcomes and broader applicability. To standardize digital workflows across centers, consensus-based guidelines on digital segmentation, replica design parameters, and print validation protocols should be developed and openly disseminated. Widespread implementation may also face regulatory hurdles, particularly regarding the classification of printed replicas as custom medical devices, which may require conformity with regional manufacturing and sterilization standards. Nonetheless, current data support the integration of digital workflows in enhancing the predictability and success of autotransplantation, particularly in anatomically challenging cases.

## Data Availability

The raw data supporting the conclusions of this article will be made available by the authors on request.
